# Systematic Comparison of the Effects of Alpha-synuclein Mutations on Its Oligomerization and Aggregation

**DOI:** 10.1371/journal.pgen.1004741

**Published:** 2014-11-13

**Authors:** Diana F. Lázaro, Eva F. Rodrigues, Ramona Langohr, Hedieh Shahpasandzadeh, Thales Ribeiro, Patrícia Guerreiro, Ellen Gerhardt, Katharina Kröhnert, Jochen Klucken, Marcos D. Pereira, Blagovesta Popova, Niels Kruse, Brit Mollenhauer, Silvio O. Rizzoli, Gerhard H. Braus, Karin M. Danzer, Tiago F. Outeiro

**Affiliations:** 1 Department of NeuroDegeneration and Restorative Research, Center for Nanoscale Microscopy and Molecular Physiology of the Brain University Medical Goettingen, Goettingen, Germany; 2 Department of Neurology, Ulm University, Ulm, Germany; 3 Georg August University, Institute for Microbiology and Genetics Dept. of Molecular Microbiology and Genetics, Goettingen, Germany; 4 Laboratório de Citotoxicidade e Genotoxicidade, Departamento de Bioquímica - Instituto de Química Universidade Federal do Rio de Janeiro, Rio de Janeiro, Brazil; 5 Instituto de Medicina Molecular, Faculdade de Medicina da Universidade de Lisboa, Lisboa, Portugal; 6 Department of Neuro and Sensory Physiology, University of Göttingen Medical Center c/o European Neuroscience Institute Göttingen, Göttingen, Germany; 7 Department of Molecular Neurology, University Hospital Erlangen, Friedrich-Alexander University Erlangen-Nürnberg, Erlangan, Germany; 8 Institute for Neuropathology, University Medical Center Goettingen, Goettingen, Germany; 9 The Department for neurosurgery at UMG and Paracelsus-Elena-Klinik, Kassel, Germany; Hebrew University of Jerusalem, Israel

## Abstract

Aggregation of alpha-synuclein (ASYN) in Lewy bodies and Lewy neurites is the typical pathological hallmark of Parkinson's disease (PD) and other synucleinopathies. Furthermore, mutations in the gene encoding for ASYN are associated with familial and sporadic forms of PD, suggesting this protein plays a central role in the disease. However, the precise contribution of ASYN to neuronal dysfunction and death is unclear. There is intense debate about the nature of the toxic species of ASYN and little is known about the molecular determinants of oligomerization and aggregation of ASYN in the cell. In order to clarify the effects of different mutations on the propensity of ASYN to oligomerize and aggregate, we assembled a panel of 19 ASYN variants and compared their behaviour. We found that familial mutants linked to PD (A30P, E46K, H50Q, G51D and A53T) exhibited identical propensities to oligomerize in living cells, but had distinct abilities to form inclusions. While the A30P mutant reduced the percentage of cells with inclusions, the E46K mutant had the opposite effect. Interestingly, artificial proline mutants designed to interfere with the helical structure of the N-terminal domain, showed increased propensity to form oligomeric species rather than inclusions. Moreover, lysine substitution mutants increased oligomerization and altered the pattern of aggregation. Altogether, our data shed light into the molecular effects of ASYN mutations in a cellular context, and established a common ground for the study of genetic and pharmacological modulators of the aggregation process, opening new perspectives for therapeutic intervention in PD and other synucleinopathies.

## Author Summary

The accumulation of aggregated proteins in the brain is common across several neurodegenerative disorders. In Parkinson's disease (PD), the protein alpha-synuclein (ASYN) is the major component of aggregates known as Lewy bodies. It is currently unclear whether protein aggregates are protective or detrimental for neuronal function and survival. The present hypothesis is that smaller aggregated species, known as oligomers, might constitute the toxic forms of ASYN. Several mutations in ASYN cause familial forms of PD. In the laboratory, artificial mutations have been designed to enable the study of the aggregation process. However, different studies relied on the use of different model systems, compromising the interpretation of the effects of the mutations. Here, we addressed this by (i) assembling a panel of 19 ASYN variants and (ii) by performing a systematic comparison of the effects of the mutations in mammalian cell models. Interestingly, our study enabled us to correlate oligomerization and aggregation of ASYN in cells. Altogether, our data shed light into the molecular determinants of ASYN aggregation, opening novel avenues for the identification of modulators of ASYN aggregation, which conceal great hopes towards the development of strategies for therapeutic intervention in PD and other synucleinopathies.

## Introduction

Alpha-synuclein (ASYN) is an abundant neuronal protein whose normal function is still elusive, but seems to be related to SNARE-complex assembly [1]. Misfolding and aggregation of ASYN in proteinaceous inclusions, known as Lewy bodies (LBs), are associated with Parkinson's disease (PD) and other neurodegenerative disorders known as synucleinopathies [2], [3]. PD is the second most common neurodegenerative disease, affecting approximately 1% of the population over 65 years of age [4], and is therefore a growing problem in the aging population. Both point mutations [5], [6], [7] and multiplications [8], [9], [10], [11] of the SNCA gene, encoding for ASYN, have been linked to autosomal-dominant forms of PD. More recently, GWAS studies identified the *SNCA* locus as a strong risk factor underlying PD [12], [13], and two additional familial mutations (G51D and H50Q) were recently identified [14], [15], [16]. The H50Q mutation is associated with late-onset parkinsonism, and the patients exhibit similar pathological features to those observed for patients carrying E46K or A53T mutations [17]. The G51D mutation is associated with early onset of disease [15].

Over the years, numerous *in vitro* and *in vivo* studies confirmed the toxic potential of both wild type (WT) and PD-linked ASYN mutants [18], [19]. *In vitro*, these ASYN mutations alter the aggregation process and interfere with oligomerization, fibril formation, and subcellular distribution [20], [21], [22]. Upon overexpression, ASYN induces aggregation and cytotoxicity [23], disrupts vesicular transport [24], [25], causes mitochondrial deficits, impairs autophagy [26], increases sensitivity to oxidative stress [27], impairs vesicle recycling, neuronal plasticity and synaptic integrity [28] as well as the folding/refolding of SNARE proteins [29]. Several animal models have also been generated based on the overexpression of either wild type or mutant ASYN, but the phenotypes reported are quite diverse [30], [31], [32].

Posttranslational modifications (PTM) in the context of PD are also controversial. The majority of ASYN in LBs isolated from PD patients is phosphorylated at serine-129 (S129) but whether this is a cause or a consequence of aggregation is unclear [33], [34], [35]. Other ASYN residues like serine-87 (S87) and tyrosines-125, -133 and -136 (Y125, Y133 and Y136) can be also phosphorylated [36]. SUMOylation, another type of PTM that modulates protein-protein interactions, affects subcellular localization, stability and solubility of target proteins [37], [38]. Engineered mutants of ASYN to prevent SUMOylation enhanced the tendency to aggregate in cell-based assays and increase cytotoxicity in dopaminergic neurons of the substantia nigra (SN), *in vivo* [38].

Although aggregation of ASYN is recognized as a central process in synucleinopathies, it is still unclear whether inclusions are toxic or protective [39]. Actually, accumulating evidence suggests ASYN oligomers may constitute the toxic species, rather than mature aggregates [40], [41]. To overcome these limitations, engineered mutation is a simple way to understand the putative effects impact of determine residues in the context of PD. Several artificial proline mutants of ASYN (A56P, A30P/A76P double mutant, and A30P/A56P/A76P triple mutant (TP) display impaired propensity to fibrilize [40]. A similar effect was reported for mutants disrupting the formation of salt bridges between β-strands of ASYN (E35K and E57K) [41], which increases the formation of oligomers when compared with WT ASYN.

Nevertheless, conflicting results obtained in different cell and animal models, and the limited existence of systematic studies comparing the behaviour of WT and ASYN mutants in the same model systems, complicate our understanding of the molecular determinants of ASYN aggregation and toxicity. Here, we conducted a systematic comparison of the effects of PD-linked and engineered ASYN mutants in two established cell-based models of ASYN oligomerization [42] and aggregation [43]. Our findings establish the effects of the different mutants studied and pave the way for the identification of genetic and pharmacological modulators of the various processes studied, opening new perspectives for the design of therapeutic strategies aimed at targeting specific steps of the ASYN aggregation process.

## Results

### Design and generation of ASYN mutants

**Figure 1:**
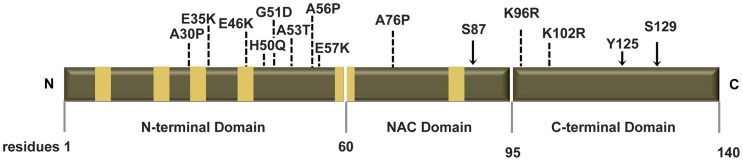
Human ASYN. Scheme representing the structure of human ASYN with the three distinct domains (N-terminal, NAC and C-terminal). Amino acid residues are indicated in the bottom. Brown bars inside protein domains represent the imperfect hexameric KTKEGV repeats. Arrows indicate the sites of phosphorylation and the broken lines show the mutated sites.

**Figure 2:**
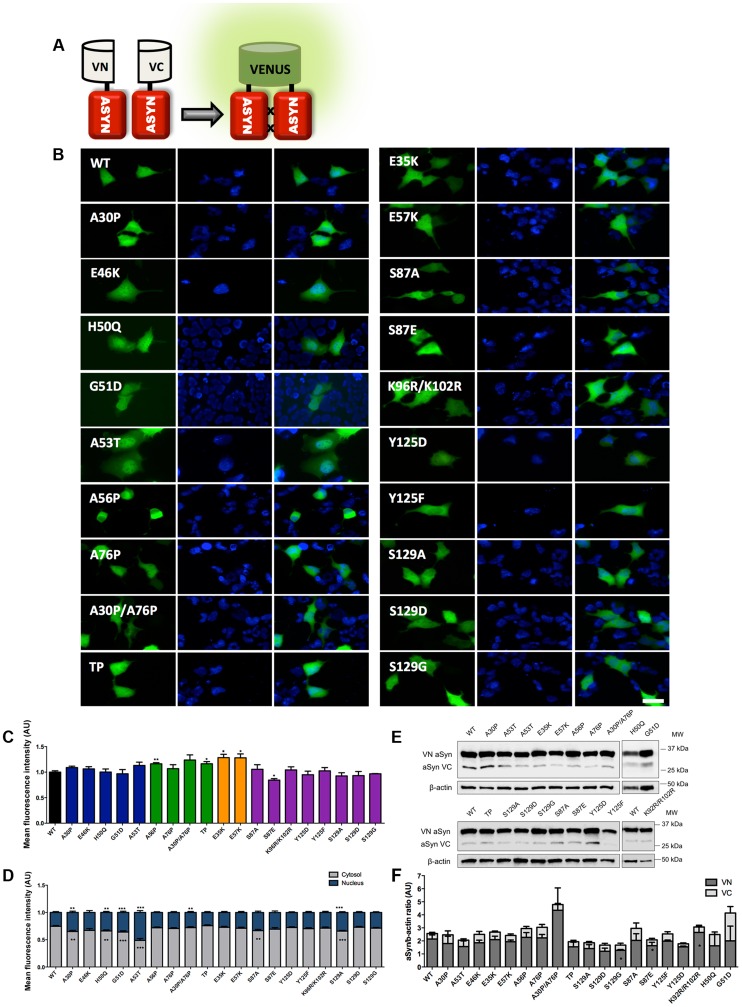
Mutations effect on ASYN oligomerization. **A. Schematic representation of Bimolecular Fluorescence Complementation assay (BiFC).** ASYN BiFC constructs in anti-parallel orientation. **B. Representative pictures of ASYN oligomerization.** HEK-293 cells overexpressing VN-ASYN and ASYN-VC constructs. The green fluorescence results from the reconstitution of the Venus fluorophore, promoted by the interaction of the proteins of interest. Scale bar: 10 µm. **C. Oligomerization efficiency.** Mean fluorescence intensity of cells expressing different ASYN mutants was assessed 24 hours post-transfection, using a microcapillary system (GuavaeasyCyte HT system). For each sample 25,000 events were counted. **D. Intracellular distribution of oligomeric ASYN.** Nuclear and cytoplasmic venus fluorescence intensities in HEK-293 cells were quantified using ImageJ. The graph demonstrates an increase in nuclear fluorescence in cells expressing ASYN mutants. For each experiment>25 cells were analysed. **E-F. Levels of ASYN.** **E**. Representative immunoblot showing the expression levels of ASYN. **F**. Immunoblot analysis of the expression levels of VN-ASYN and ASYN-VC from all the mutations studied in HEK-293 cells. Student's *t* test (*p<0.05, **p<0.01, ***p<0.001). n = 3.

**Figure 3:**
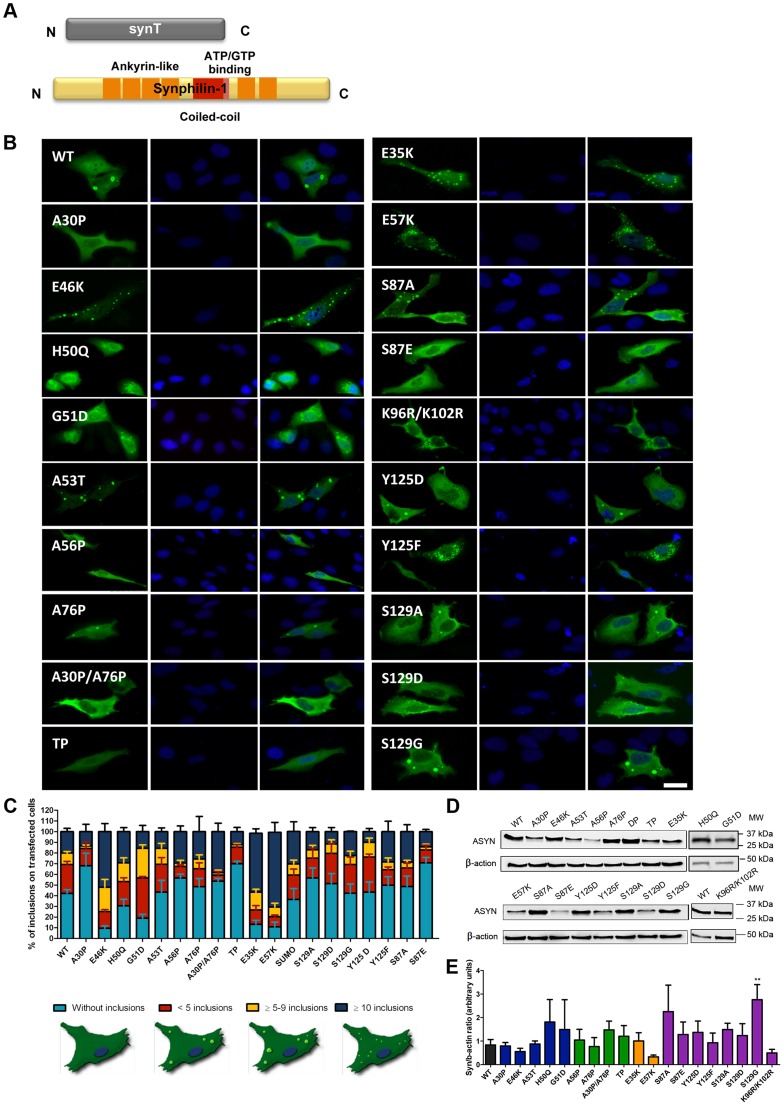
ASYN mutation effects in the inclusion formation. **A. Constructs used in the aggregation model.** This model consists of co-expressing SynT together with synphilin-1. **B. Inclusion pattern in H4 cells.** Different SynT mutants resulted in the formation of distinct inclusion formation in human H4 cells. Scale bar: 10 µm. **C. Inclusion quantification.**>50 cells were scored per experiment and classified in different groups according to the pattern of inclusions. Representative cells were drawn to show type of inclusions present in each categories. Lysine mutants (E35K, E57K) increase the percentage of cells with inclusions and the number of inclusions per cell, whereas A30P and proline mutants reduce percentage of cells with inclusions and also the number of inclusions per cell. **D-E. Levels of ASYN.** Immunoblot analysis of the expression levels of ASYN. Student's *t* test (*p<0.05, **p<0.01, ***p<0.001). n = 3.

To investigate the molecular determinants of ASYN oligomerization and aggregation in a cellular context, we used site-directed mutagenesis to generate a panel of 19 ASYN point mutants including five mutations associated with familial PD (A30P, E46K, H50Q, G51D and A53T) and others known to interfere with different aspects of ASYN biology (Fig. 1 and Table S1). Then, we analysed the behaviour of each mutant in established paradigms of ASYN oligomerization (Fig. 2A) or aggregation (Fig. 3A).

### Effect of mutations on ASYN oligomerization

In order to assess the effect of ASYN mutations on oligomerization, we used a variant of the Bimolecular Fluorescence Complementation (BiFC) assay we previously described [42], based on the reconstitution of functional Venus fluorescent protein promoted by the interaction between, at least, two ASYN molecules, that enables us to directly visualize the formation of ASYN dimeric/oligomeric species (hereforth referred to as oligomeric species for simplicity) in living cells (Fig. 2A) [42]. We have previously demonstrated that the efficiency of the ASYN BiFC assay is identical in different cell lines, including HEK cells [42]. Using epifluorescence microscopy we found that, as expected, all the ASYN variants formed oligomers in HEK cells (Fig. 2B and C). One striking observation was that the PD-linked mutant A53T promoted the strongest increase (∼50%) in the accumulation of ASYN oligomers in the nucleus (Figure 2B and D).

To compare the extent to which different ASYN variants promoted oligomerization we used flow cytometry to measure the fluorescence intensities of cells expressing the different mutants. We observed an increase in fluorescence intensity for proline and lysine mutants. This increase was around 16.3% for A56P mutant, and 16.5% for the TP mutant (Fig. 2C). In the case of A30P/A76P double mutant (DP) and single A76P mutant we observed a trend towards an increase in fluorescence intensity, but the increase was not statistically significant. Likewise, we found a 28.4% increase in oligomerization for the E35K mutant, and 28.1% for the E57K mutant. In contrast, we found that the S87E mutant, mimicking phosphorylation at S87, reduced oligomerization by ∼16%. To investigate whether these effects were explained by differences in the levels of ASYN, we performed immunoblot analysis. We found that the levels of VN-ASYN decreased for almost all mutants (statistically significant for G51D, S129D and S87A) and increased for H50Q, A30P/A76P and K96R/K102R (Fig. 2E, F). Interestingly, there was no correlation between the levels of the mutants and the fluorescence signal, suggesting the effects observed were intimately correlated with the effects of the mutations on oligomerization, and not due to differences in the levels of expression of the various ASYN mutants.

### Effect of mutations on ASYN aggregation

In parallel, we asked whether the selected mutations altered ASYN inclusion formation. For this, we took advantage of an established paradigm of ASYN aggregation based on the co-expression of SynT and synphilin-1, an ASYN-interacting protein that is also present in LBs (Fig. 3A) [43]. As previously established, human neuroglioma cells (H4) were co-transfected with plasmids encoding each of the SynT variants and synphilin-1 and inclusion formation was assessed 48 hours post-transfection. Since the inclusion pattern was heterogeneous, we defined four categories (cells without inclusions, with <5 inclusions, with more than 5 and less than 9 inclusions, or ≥10 inclusions) in order to obtain a more precise assessment of the effects of the mutations. For the PD-linked mutants, we found that A30P increased the percentage of cells without inclusions to ∼70% when compared to WT ASYN. In contrast, the E46K and G51D mutations dramatically increased the percentage of cells with inclusions to ∼90% and ∼80%, respectively (Fig. 3B, C and Fig. S1).

In the case of the proline mutants, we observed that all four mutants reduced the percentage of cells displaying inclusions (Fig. 3B, C and Fig. S1).

For the E35K and E57K mutants, the number and the size of the inclusions varied. Both mutants promoted an increase to ∼70–80% of cells with inclusions (Fig. 3B, C and Fig. S1). While we predominantly observed the presence of small inclusions with the E57K mutant, we detected a mix of small and larger inclusions with the E35K mutant (Fig. 3B).

We also investigated the effect of phosphorylation on ASYN inclusion formation. For this, we screened mutants that block (S87A, Y125F, S129A and S129G) or mimic (S87E, Y125D and S129D) phosphorylation. We found that mimicking phosphorylation on S87 resulted in a marked decrease in the number of inclusions per cell, with ∼80% of the transfected cells displaying no inclusions (p<0.01 Fig. 3C, Fig. S1A). No significant effect was observed with the S87A mutant, suggesting the S87 may normally exist, at least in our cell model, mostly unphosphorylated. Also, no significant differences were observed for Y125D, S129A, S129G, or S129D mutants, when compared to WT SynT. However, we found the Y125F mutation and SUMOylation-deficient mutant (K96R/K102R) mutation induced an altered inclusion pattern, with the accumulation of inclusions of different sizes, similar to that observed with the lysine mutant.

Immunoblot analysis showed that the levels of ASYN varied depending on the particular mutant being expressed, but we only found a significant increase in the levels of the S129G mutant (Fig. 3D and E). Interestingly, we found a trend towards a decrease in the levels of mutants that promoted accumulation of inclusions or changed the size of the inclusions (E46K, E57K and K96R/K102R).

**Figure 4:**
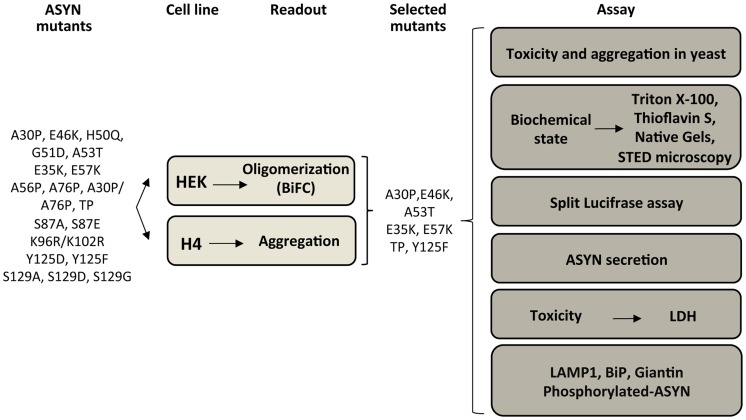
Experimental design. WT and ASYN mutants were subjected to a variety of assays as depicted in the schematic.

Based on the results obtained in the oligomerization and aggregation paradigms, we decided to focus on seven ASYN mutations (A30P, E46K, A53T, E35K, E57K, TP and Y125F) that had the most pronounced effects for subsequent analysis (Table S2 and Fig. 4). We examined these selected ASYN mutants using different assays, including toxicity measurements, biochemical analysis of ASYN, ASYN secretion, degradation pathways, and Golgi and ER stress (Fig. 4), in order to obtain detailed information on the cellular effects of specific types of ASYN accumulations.

### ASYN toxicity and aggregation in yeast

**Figure 5:**
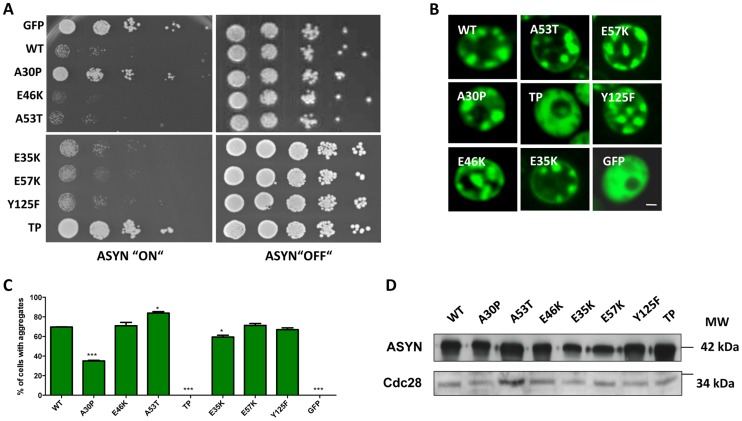
High-copy expression of α-synuclein-GFP variants in yeast. **A**. Yeast cells expressing GAL1-driven ASYN-GFP variants from 2 µ plasmids were spotted in 10-fold dilutions on selection plates containing 2% glucose (control) or 2% galactose. After incubation for 3 days at 30°C the plates were photographed. Expression of GFP from the same promoter was used as a control. **B. Live-cell fluorescence microscopy of yeast cells expressing ASYN-GFP.** Yeast cells, pre-grown to mid-log phase, were induced in galactose-containing medium and examined for aggregates at 6 hours of induction. GFP-expressing cells were used as control. Scale bar: 1 µM. **C. Aggregate quantification of yeast cells, expressing ASYN-GFP.** For each strain, the number of cells displaying cytoplasmic foci is presented as percent of the total number of cells counted. For quantification of aggregation at least 300 cells were counted per strain and per experiment. GFP-expressing cells were used as a control. Student's *t* test (*p<0.05, **p<0.01, ***p<0.001). **D. Protein levels of ASYN-GFP variants.** Expression of ASYN-GFP variants was induced for 6 hours in galactose-containing medium. Equal amounts of crude protein extracts were used for Western analysis with anti-ASYN antibody and anti-cdc28 antibody as a loading control. n = 2.

We started by investigating the toxicity of the selected variants of ASYN by taking advantage of the budding yeast as a model of synucleinopathies, as previously described [23], [44]. In conditions where the expression of ASYN was induced, using galactose-containing media, we found that almost all mutations induced toxicity similar to WT ASYN (Fig. 5A). In line with the observation in H4 cells, the TP mutant did not form inclusions and the A30P mutant strongly impaired inclusion formation (Fig. 3B-C and 5B-C). Neither of these mutants was toxic in yeast (Fig. 5A) [23], [44]. Importantly, we found no significant differences in the levels of expression of all variants tested, ruling out the possibility that toxicity and/or inclusion formation were due to differences in the levels of expression of ASYN (Fig. 5D).

### Characterization of ASYN species

**Figure 6:**
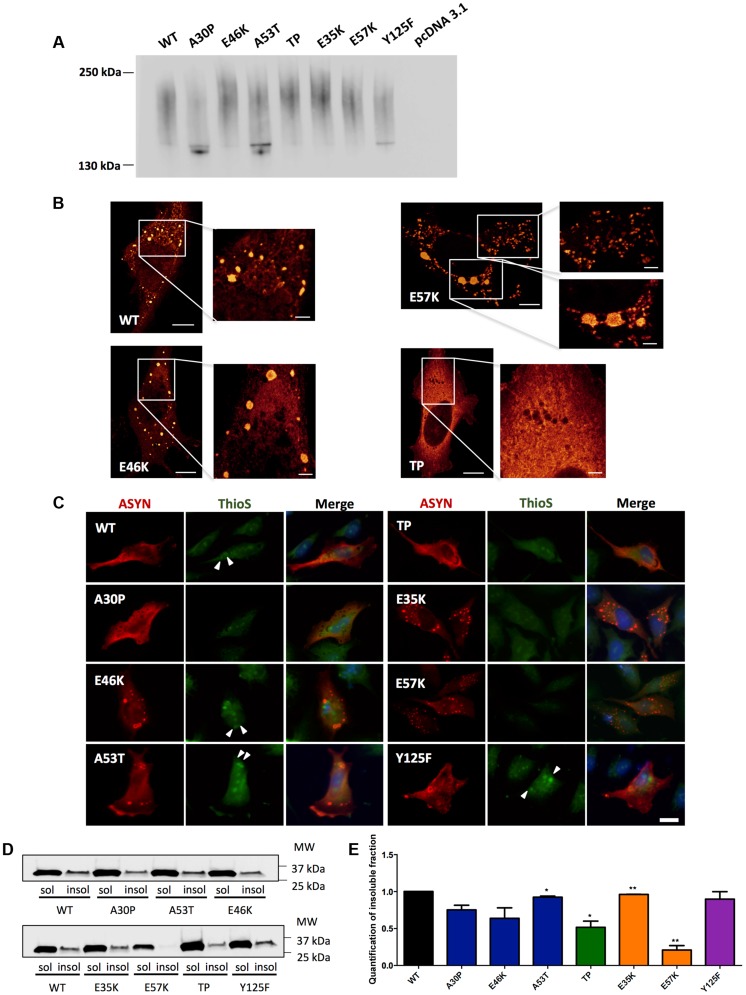
ASYN biochemical state. **A. Native Gels.** Immunoblot analysis of native PAGE of cells transfected with the BiFC constructs in HEK 293 cells. Smears indicate the presence of oligomeric species of ASYN with different sizes. n = 2. **B. STED microscopy.** Selected mutants were imaged in order to characterize the fine structure of the inclusions. **C. Thioflavin S staining.** H4 cells expressing selected SynT mutants were incubated with ThioS in order to reveal beta sheet-rich structures. Some of the inclusions display amyloid-like properties, with increased staining in the inner part of the inclusions, indicated with arrow heads (▸). Scale bar: 10 µm. **D-E**. **Triton X-100 solubility assay and quantification.** H4 cells show that all mutants form detergent insoluble species. Student's *t* test (*p<0.05, **p<0.01, ***p<0.001). n = 2. Quantification of insoluble fraction shows a decrease in TP and E57K mutants.

To further assess the biochemical nature of the ASYN species visualized by the BiFC assay, we employed non-denaturing polyacrylamide gel electrophoresis (native-PAGE). Immunoblot analysis showed a smear, which is indicative of the accumulation of oligomeric species of various sizes (Fig. 6A).

In order to characterize the structure of the ASYN inclusions formed in H4 cells, we used stimulated emission depletion (STED) super resolution microscopy (Fig. 6B). STED provided unprecedented access to the fine structure of the ASYN inclusions in the cytoplasm. We focused on selected mutants that displayed extreme patterns of aggregation (Fig. 3) and found that both smaller and larger inclusions are highly compact. For the TP mutant, only diffuse signal was detected, confirming the inability of this mutant to accumulate in inclusions that can be resolved by light microscopy techniques (Fig.6B).

It is widely established that LBs are primarily composed of amyloid filaments of ASYN [2]. To determine whether the inclusions formed by the different ASYN mutants were composed of amyloid-like fibrils, we used thioflavin S (thioS), a dye that binds specifically to amyloid-like structures [26], [45]. We verified that large inclusions formed by WT or mutant ASYN stained positive for thioS, whereas small inclusions did not (Fig. 6C). Also, thioS stained the inner part of the inclusions (marked with arrow head) (Fig. 6C), suggesting the accumulation of mature amyloid-like structures in the inner part of the inclusions. To further characterize these different types of ASYN aggregates, we assessed the detergent solubility of the inclusions formed by the selected ASYN mutants. Interestingly, we observed that TP and E57K accumulated a smaller fraction of Triton X-100 insoluble ASYN species (Fig. 6D and E).

### Intra- and extracellular partitioning of ASYN oligomers

**Figure 7:**
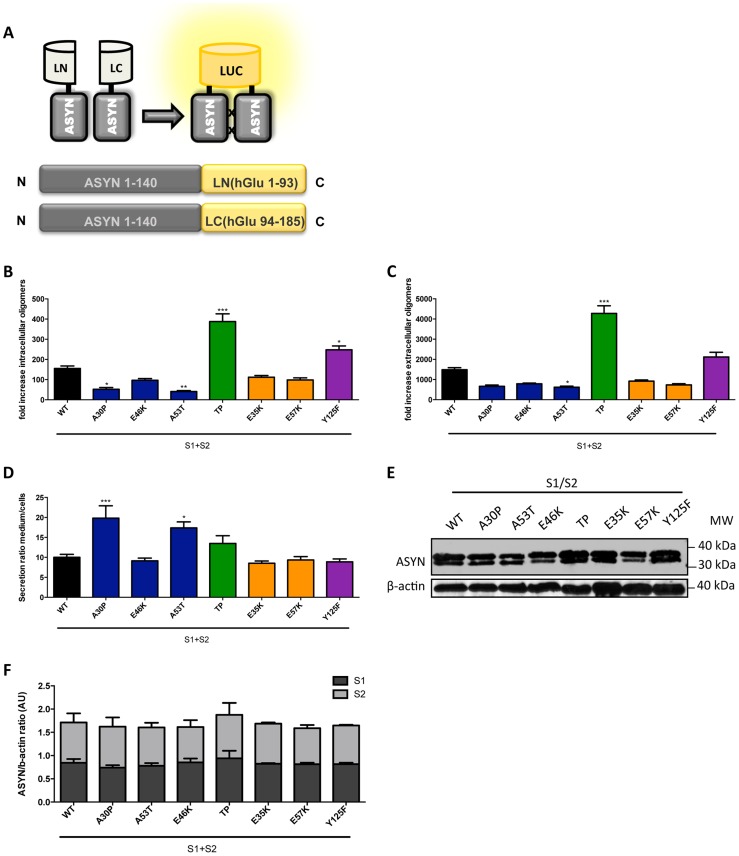
ASYN bPCA. **A. Schematic representation of the ASYN bPCA constructs.** Non-bioluminescent halves of humanized Gaussia luciferase (hGLuc) were fused to ASYN monomers. **B-C**. Intact cells (intracellular) and medium (extracellular) from H4 cells co-transfected with S1 and S2 were assayed for luciferase activity 48 hours post-transfection. Intracellular (**B**) and extracellular (**C**) TP displayed a 3-fold increase in luciferase activity compared to WT. n = 12. Student's *t* test (*p<0.05, **p<0.01, ***p<0.001) **D**. Ratio of luciferase activity in media compared to cells was expressed. n = 12, Student's *t* test (*p<0.05, **p<0.01, ***p<0.001) **E-F. Levels of ASYN.** Immunoblot analysis of the expression levels of ASYN showing similar levels. n = 3.

To assess the effect of the selected mutations on the distribution of ASYN oligomers inside and outside cells, we used a previously described bioluminescent protein complementation assay (bPCA) that enables the detection of oligomeric species with great sensitivity [46]. In this assay, reconstitution of *Gaussia princeps* luciferase activity upon ASYN oligomerization was used as a readout [47] (Fig. 7A). Consistent with the results obtained with the Venus-based BiFC assay (Fig. 2A), we detected reconstitution of luciferase activity with all mutants tested. However, we observed a strong increase in intracellular (Fig. 7B) and extracellular (Fig. 7C) luciferase activity with the TP and Y125F ASYN mutants when compared to WT ASYN. This indicates that not only these mutations are able to promote increased formation of oligomers inside cells, but also in the extracellular space.

To determine if these mutants also promoted the release of oligomeric species we calculated the ratio of luciferase activity in the media compared to that in cells. Interestingly, we found that familiar mutants A30P and A53T showed an increased ratio of luciferase activity outside versus inside cells suggesting that these mutants also promote the secretion of ASYN oligomers (Fig. 7D). We confirmed these differences were not simply due to the accumulation of increased levels of mutant ASYN, since the levels of expression were identical for all mutants tested (Fig. 7E-F).

### ASYN secretion is inversely correlated with toxicity

ASYN is a cytosolic protein, but recent studies detected both monomeric and oligomeric forms of ASYN in human cerebrospinal fluid and plasma at low nanomolar concentrations, in both PD and control individuals [48], [49].

**Figure 8:**
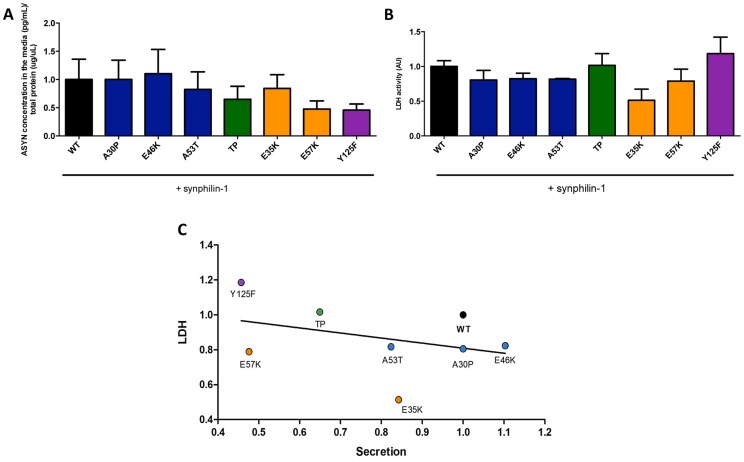
ASYN secretion is inversely correlated with toxicity. **A. Secretion of ASYN. B. Toxicity measurements.** Medium from H4 cells were collected to determine the secretion and the percentage cytotoxicity for each mutant. To measure the release of ASYN, an ELISA assay was performed. Using the same media we also measured the release of lactate dehydrogenase as a measure of cytotoxicity. We observed that these values were inversely correlated with those obtained in the release/secretion experiments. A decrease trend particularly for TP and Y125F detected in terms of secretion, was higher in toxicity. n = 3. **C. Correlation between Secretion and Toxicity.** The graph shows the inverse trend in secretion and toxicity.

To complement the observations with the bioluminescence complementation assay, we asked whether the selected ASYN mutants were differentially released from cells using the aggregation paradigm described above (Fig. 3A). In this case, we measured the levels of ASYN in the culture medium using a highly sensitive electrochemiluminescence-based immunoassay [50]. We detected extracellular ASYN with all variants tested (Fig 8A).

Next, to demonstrate that the release of ASYN was not caused by membrane leakage from unhealthy or dying cells, we performed LDH toxicity measurements in the same media. In fact, in the aggregation paradigm, we observed an inverse correlation between ASYN release and cytotoxicity, where the TP and Y125F mutants appeared as the most toxic forms (Fig. 8B and C).

### Effect of ASYN mutants on lysosomal degradation

**Figure 9:**
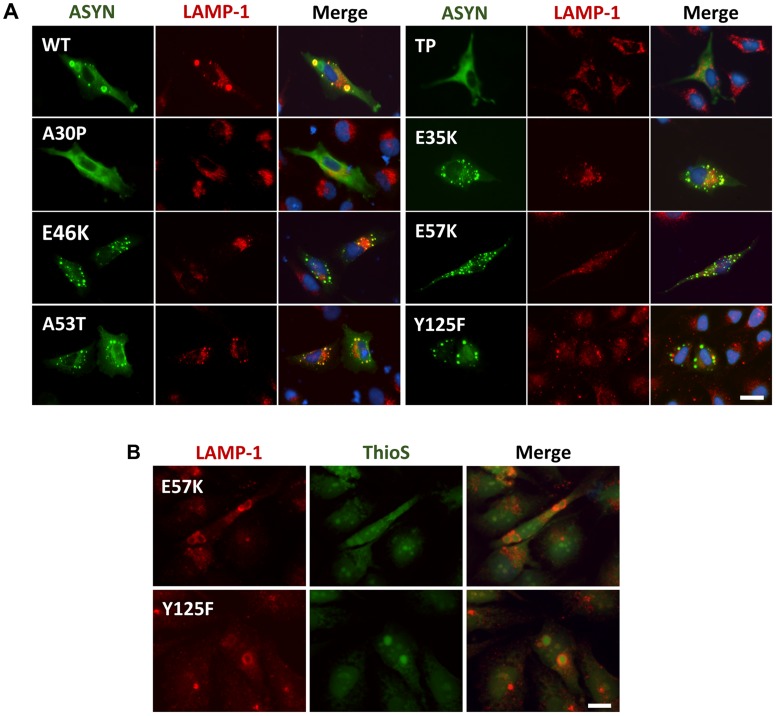
ASYN partially co-localizes with endosomes/lysosomes. **A. Immunocytochemistry analysis of H4 cells expressing selected ASYN mutants.** Partial co-localization of ASYN and LAMP1 suggests interplay between lysosomal degradation and ASYN inclusion formation. **B. E57K and Y125F inclusions co-localize with lysosomal marker LAMP-1.** We detected the presence of endosomes/lysosomes surrounding the aggregates in E57K and Y125F. This indicates that, maybe this could be the preferential via for degradation for these mutations. Scale bar: 10 µm.

Knowing that ASYN is predominantly degraded by lysosomal pathways and, therefore, requires intact lysosomal function, we used lysosomal associated membrane protein 1 (LAMP-1) as a marker to establish the relationship with aggregation formation. We observed that LAMP-1 partially co-localized with ASYN inclusions (Fig. 9A), suggesting that at least some types of inclusions might be degraded in lysosomes. Interestingly, we observed that some inclusions formed by two of the mutations that primarily accumulated thioS-negative inclusions (E57K and Y125F, Fig. 6B) stained positive for LAMP-1 at the periphery (Fig. 9B), reinforcing the idea that specific types of ASYN inclusions are degraded in lysosomes.

### Golgi fragmentation and ER stress

**Figure 10:**
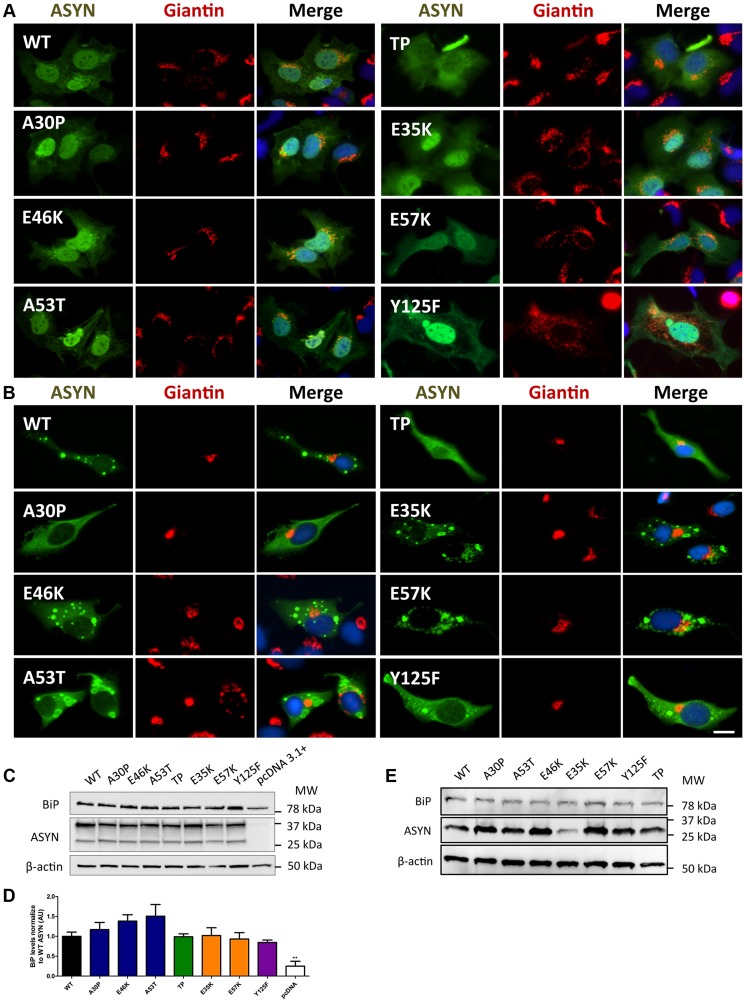
A-B morphology analysis of Golgi apparatus. The morphology of the Golgi apparatus in>50 cells was analysed and quantified. We observed that, in the BiFC assay, E35K and E57K mutants displayed increased Golgi fragmentation (**A**). In the aggregation model, Golgi morphology appeared normal, displaying a compact appearance near the nucleus **(B). Levels of BiP in the oligomerization assay (C) and in the aggregation model (E), assessed by immunoblot analysis and respective quantifications (D and E).** n = 3. Student's *t* test (*p<0.05, **p<0.01, ***p<0.001).

Given that fragmentation of Golgi apparatus (GA) has been described in several neurodegenerative diseases [51], [52], [53], we next investigated the cellular consequences of the accumulation of ASYN oligomers or inclusions on this organelle. For this, we examined the morphological integrity of the GA using fluorescence microscopy of cells immunostained for Giantin, an endogenous transmembrane protein of the cis and medial Golgi complex (Fig. 10). We defined three types of Golgi structures (non-fragmented, diffuse and fragmented). In general, we observed that in the ASYN oligomerization model there was an increased percentage of cells displaying fragmented Golgi, in comparison to what was observed in the aggregation model (Fig.10 A-B and Fig. S2). In particular, we found a statistically significant increase in the percentage of cells displaying fragmentation of the GA for the E35K and E57K mutants (Fig. 10A and Fig. S2A). In the ASYN aggregation paradigm, the GA displayed normal compact morphology near the nucleus (Fig. 10B and Fig. S2B).

Recent studies showed that endoplasmic reticulum (ER) stress, together with deficient protein degradation, plays a crucial role in the death of dopaminergic cells [54]. Under ER stress conditions, BiP is upregulated and preferentially binds to misfolded proteins in the ER [55]. We observed that oligomeric forms of ASYN promoted an increase in the levels of BiP (E46K and A53T mutants)(Fig.10C and D) whereas no differences were detected in the aggregation paradigm (Fig.10 E).

Taken together, these results suggest oligomeric forms of ASYN are more capable of promoting Golgi fragmentation and ER stress than aggregated forms.

### Effect of mutations on ASYN phosphorylation

Accumulating evidence implicates phosphorylation on ASYN aggregation and toxicity [56]. However, it is unclear whether mutations in ASYN affect the typical pattern of phosphorylation. Using the oligomerization assay, we investigated whether ASYN was differentially phosphorylated on S129 (Fig. S3). Interestingly, we detected a significant increase in S129 phosphorylation in the E35K mutant, and only a trend towards an increase for the familial mutants and E57K (Fig. S3). The results shows that phosphorylation of ASYN on S129 is altered in the context of specific mutations known to affect the aggregation of the protein.

## Discussion

Given the central role of ASYN in PD and other synucleinopathies, and the uncertainty about the precise molecular mechanisms leading to neurodegeneration, we sought to take advantage of various cellular systems in order to systematically compare a set of ASYN mutants according to their effects on different cell functions.

**Figure 11:**
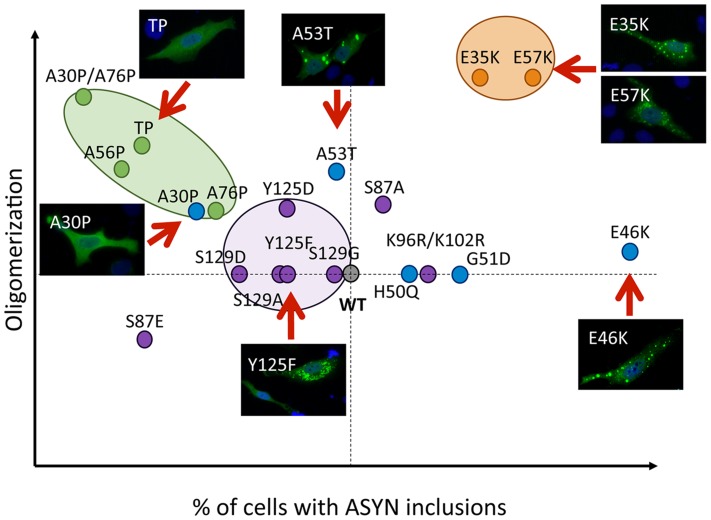
Correlation between the effects of ASYN mutations on oligomerization and inclusion formation. The graph depicts how mutations affect oligomerization and inclusion formation, enabling the selection of mutants with different effects. Values were attributed to ASYN mutations according to the results from the two models (oligomerization and inclusion formation) using WT ASYN as reference (center of the graph).

Our systematic analysis enabled us to directly compare the effects of the selected mutations in terms of oligomerization and aggregation (Fig. 11). We observed that all 19 mutations tested enabled the formation of ASYN oligomers, as assessed by the BiFC system and biochemical methods. Interestingly, we found the different mutants resulted in the accumulation of different types of inclusions.

While we did not detect significant differences in the oligomerization induced by familial PD mutations, the A30P mutant displayed a reduced propensity to form inclusions, in contrast to the E46K and G51D mutants, which enhanced inclusion formation when compared with WT ASYN. The fact that we observed a decrease in the number of inclusions with the A30P might be related to the long-range contacts between the N and C-termini, shielding the central domain, which is known to promote aggregation, and reducing the formation of the same types of inclusions observed with WT or other mutants. On the other hand, the robust increase of inclusion formation observed with the E46K mutant might be due to the location of the mutation within the KTKEGV repeats that are involved in alpha-helix formation [57]. The charge difference introduced by the mutation could enhance the destabilization of the protein structure, leading to an increased propensity to aggregate, as reported previously [57]. Recent studies showed that the G51D mutation attenuates aSyn aggregation *in vitro* [58], [59]. However, we observed a different trend in our cell models, where G51D increased the number of aSyn inclusions per cell, as observed with the E46K mutant. This might be explained by differences in the cellular environment in the different models, but might also be due to the presence of synphilin-1 in the particular model we used for aSyn aggregation. The same might apply to the H50Q mutant since, in contrast to other studies, we did not observe any difference in comparison to WT aSyn [59]. For the A53T mutant we found increased presence of oligomeric species in the nucleus, but no differences in terms of the aggregation pattern. Thus, additional studies on the effect of ASYN in the nucleus will be important.

The use of engineered mutants enables the exploration of the structural determinants of ASYN physiology in the cell. Artificial proline mutations were designed to impair fibrillization *in vitro* and promote the formation of soluble oligomers [40]. Indeed, in our cell models, the proline mutants resulted in increased oligomerization (Fig. 2B and C) and lowered the propensity to form inclusions. In particular, the TP mutant increased oligomerization as assessed by BiFC and bPCA, and reduced inclusion formation. These observations were also confirmed in yeast cells, where the A30P formed fewer inclusions, and the TP mutant completely blocked inclusion formation. However, in contrast to what was observed in primary neurons, worms, and in flies [40], the proline mutants failed to promote increased cytotoxicity in yeast cells. One possibility is that yeast process the species formed by the proline mutants in a distinct manner, explaining the lower levels of toxicity.

The lysine mutants (E35K and E57K) were designed to disrupt salt bridges between the β-strands of ASYN and to interfere with its binding to lipid membranes [41]. We found both mutants increased ASYN oligomerization and promoted distinct inclusion patterns. The lack of thioS staining suggests the inclusions may represent either off-pathway or immature species that cannot proceed towards the formation of mature amyloid-like inclusions. Interestingly, in cells where we could detect inclusions formed by the E57K mutant, these appeared surrounded by LAMP1, suggesting they were targeted to lysosomal degradation.

Post-translational modifications (PTMs) are important modulators of the structural and functional properties of proteins in health and pathological conditions. Several lines of evidence suggest that phosphorylation of ASYN may play an important role in regulating its aggregation, fibrillogenesis, Lewy body formation, and neurotoxicity *in vivo*. In addition, it appears that, *in vivo*, less than 5% of ASYN is normally phosphorylated [60] and that this occurs predominantly in the C-terminus (S129 and Y125) but also in the NAC domain (S87). However, there is still no consensus on the effects of phosphorylation due to existing contradictory results [56], [61], [62], [63]. S87 is located in the NAC region, which is crucial for ASYN aggregation and fibrillogenesis [64] and is also the region involved in interactions with other proteins [62]. Our study supports the importance the NAC domain in the formation of ASYN inclusions, as the S87E phosphomimic mutant induced different effects than those observed with the S87A mutant (Fig. 3B and C). This is in line with *in vitro* experiments where the S87E mutant inhibits ASYN aggregation [62]. Again, this effect on the formation of cytoplasmic inclusions might be attributed to changes in the conformation when binding to membranes, as other studies suggested [63]. Moreover, a recent study showed that S87E ASYN reduces aggregation and is less toxic [62].

Detecting significant levels of Y125-P in ASYN in human brain tissues has proven difficult [65]. Our results indicate, in the context of living cells, Y125-P ASYN exhibits similar aggregation properties to WT ASYN, in accordance with what was observed in other systems [65]. We also observed the accumulation of small inclusions for the Y125F mutant that were similar to those formed by the lysine mutants.

Recently, it was shown that when SUMO acceptor sites in ASYN (K96R/K102R) are modified, SUMOylation is strongly impaired, leading to increased inclusion formation and toxicity [38]. In our study, we observed only a 10% increase in the percentage of cells displaying inclusions and, interestingly, we observed the accumulation of smaller inclusions. These small inclusions promoted by several mutants tested (E35K, E57K, Y125F and SUMOylation mutants, Fig.3B) might represent intermediate species in the aggregation process of ASYN that fail to mature and may, therefore, lead to proteasomal impairment. To gain insight into the cellular consequences of different types of ASYN accumulations, we selected representative mutations for additional studies.

ASYN overexpression and/or aggregation can affect the secretory pathway. One of the consequences observed is the disruption of the Golgi and impairment ER-to-Golgi trafficking. Fragmentation of this organelle has been reported in neurodegenerative disorders [51], [52], including PD [53], and it was shown that it occurs in the cells accumulating prefibrillar ASYN aggregates [66]. Indeed, we observed the same trend in our study, where increased Golgi fragmentation was observed in the oligomerization model, particularly with E35K and E57K ASYN. Furthermore, we observed increased levels of BiP with familial mutants of ASYN in the oligomerization model. This was not detected in the aggregation model, further supporting the concept that oligomers are detrimental and disturb proteostasis by affecting the normal intracellular trafficking.

Autophagy is a catabolic process that is involved in the control of cellular damage in response to genetic perturbations, aging, and/or environmental toxins. Our study also underscores the interplay between ASYN inclusion formation and autophagy since we found the lysosomal marker LAMP-1 surrounding and concealing mature inclusions, as judged by thioS staining (Fig. 9B). Again, this reinforces the idea that aggregates per se might not be directly harmful to cells but, instead, might constitute an effort for cells to remove abnormal proteins from the cytoplasm.

Altogether, the systematic assessment of the effects of different ASYN mutations on its oligomerization and aggregation in cellular models allowed us to address, for the first time, the effect of the selected mutations on a panel of readouts that reflect important aspects of the biology/pathobiology of the protein. While additional studies using alternative models will be important to further dissect the effects of mutations, our study establishes the foundation for testing hypotheses that may open novel opportunities for the development of therapeutic strategies for synucleinopathies.

## Materials and Methods

### Primer design

The primers were designed according to the manufacturer's instructions using the QuickChange Primer Design Program and the web-based program Primer X (Table S1).

### Generation of the mutant ASYN constructs

Site-directed mutagenesis using QuickChange II Site-Directed Mutagenesis Kit (Agilent Technologies, SC, USA) was performed following the manufacturer's instructions. Mutagenesis were performed in the plasmids encoding the ASYN-Venus BiFC system [42] or SynT [43] and confirmed by DNA sequencing. Also, fusion constructs ASYN-hGLuc1 (S1) and ASYN-hGLuc2 (S2) were generated as described previously [42].

Yeast plasmids expressing GFP (pME3759), human wild-type ASYN-GFP (pME3763), A30P-GFP (pME3764), A53T-GFP (pME3765) and TP-GFP (pME3942) from galactose-inducible promoter (GAL1) were described previously [67]. Plasmids harboring E46K-GFP (pME4085), E35K-GFP (pME4086), E57K-GFP (pME4087) and Y125F-GFP (pME4088) were generated by site-directed mutagenesis using the same primers as above. Plasmid pME3763 was used as a template for generation of the desired amino acid substitutions.

### Cell culture

Human neuroglioma cells (H4) were maintained in Opti-MEM I Reduced Serum Medium (Life Technologies- Gibco, Carlsbad, CA, USA) and Human Embryonic Kidney 293 (HEK) cells were grown in Dulbecco's Modified Eagle Medium (DMEM, Life Technologies- Invitrogen, Carlsbad, CA, USA). Both media were supplemented with 10% Fetal Bovine Serum Gold (FBS) (PAA, Cölbe, Germany) and 1% Penicillin-Streptomycin (PAN, Aidenbach, Germany). The cells were grown at 37°C in an atmosphere of 5% CO_2_.

### Cell transfection

#### HEK cells

The cells were plated in 24-well plates (Costar, Corning, New York, USA) the day before. Thirty minutes before the transfection the cells were incubated in Opti-MEM (Life Technologies-Invitrogen, Carlsbad, CA, USA) and subsequently transfected with equimolar amounts of the plasmids using Metafectene (Biotex, Munich, Germany) according to the manufacturer's instructions. Five hours after transfection, the medium was replaced. Twenty-four hours after transfection the cells were collected or stained for further analysis.

#### H4 cells

Twenty-four hours prior to transfection, the cells were plated in 35 cm dish (Ibidi, Munich, Germany) or in 12-well plates (Costar, Corning, New York, USA). Equal amount of the plasmids encoding ASYN and synphilin-1 were transfected using FuGENE6 Transfection Reagent (Promega, Madison, USA) in a ration of 1∶3 according to the manufacturer's instructions. Forty-eight hours after the transfection, the media were collected and frozen for further experiments. Additional, cells were subjected to immunocytochemistry, for studying ASYN inclusions.

### Gaussia luciferase protein-fragment complementation assay

For the bioluminescence complementation assay with ASYN-hGLuc1 (S1) and ASYN-hGLuc2 (S2) constructs, the cells were transfected as described above and conditioned media was collected 48 hours post-transfection and centrifuged for 5 minutes at 3000 g to eliminate floating cells before being used.

Forty-eight hours after transfection, culture media was transferred to a new 96 well plate (Costar, Corning, NY, USA). Cells were washed with PBS and replaced with serum- and phenol-red free media. Luciferase activity from protein complementation was measured for conditioned media and live cells in an automated plate reader at 480 nm with a signal integration time of 2 seconds following the injection of the cell permeable substrate, Coelenterazine (20 µM) (PJK, Kleinblittersdorf, Germany).

### Yeast cell culture conditions

Yeast strain W303-1a (*MATa; ura3-1; trp1Δ 2; leu2-3,112; his3-11,15; ade2-1; can1-100*) was used for transformation performed by standard lithium acetate protocol. All strains were grown in Synthetic Complete medium lacking uracil (SC-Ura), supplemented with 2% raffinose or 2% galactose. ASYN expression was induced by shifting yeast cells cultured overnight in raffinose to galactose medium (OD600  = 0.1).

Overnight cultures of yeast strains were grown in SC-Ura medium containing 2% raffinose. For induction of the GAL1 promoter, cells were inoculated in SC-Ura medium containing 2% galactose to an OD600  = 0.1 and incubated for 6 h. Cell extracts were prepared and the protein concentrations were determined with a Bradford assay. Immunoblotting was performed following standard procedures using anti-ASYN monoclonal antibody (AnaSpec, CA, USA) or Cdc28 polyclonal antibody (Santa Cruz Biotechnologies, Santa Cruz, CA, USA) as a loading control.

### Spotting assays

To analyse cell growth on solid media, cultures were grown to mid-log phase in SC-Ura medium containing raffinose. Cells were normalized to equal densities, serially diluted 10-fold starting with an OD600 of 0.1, and spotted on SC-Ura plates containing either 2% glucose or 2% galactose. After 3 days incubation at 30°C the plates were photographed.

### Fluorescence microscopy and quantifications of yeast cells

Yeast cell cultures were grown in SC-Ura medium containing 2% raffinose until mid-log phase and transferred to SC-Ura medium supplemented with 2% galactose. Expression was induced for 6 h and fluorescent images were obtained with Zeiss Observer. Z1 microscope equipped with CSU-X1 A1 confocal scanner unit (YOKOGAWA), QuantEM:512SC (Photometrics) digital camera and SlideBook 5.0 software package (Intelligent Imaging Innovations). For quantification of aggregation at least 300 cells were counted per strain and per experiment. For each strain, the number of cells displaying cytoplasmic foci was reported to the total number of cells counted and displayed as percentage on a column chart.

### Immunocytochemistry

Twenty-four or forty-eight hours after transfection, cells (HEK or H4) were washed with PBS and fixed with 4% paraformaldehyde (PFA) for 10 minutes at room temperature (RT), followed by a permeabilization step with 0.5% Triton X-100 (Sigma-Aldrich, St. Louis, MO, USA) for 20 minutes at RT. After blocking in 1.5% normal goat serum (PAA, Cölbe, Germany)/DPBS for 1 hour, cells were incubated with primary antibody. Primary antibodies used were: mouse anti-ASYN (1∶1000, BD Transduction Laboratory, New Jersey, USA) or rabbit anti-ASYN (1∶1000, Abcam, Boston, USA), rabbit anti-LAMP-1 (1∶1000, Abcam, Boston, USA), anti-Giantin (1∶1000, Abcam, Boston, USA) for 3 hours or overnight and secondary antibody (Alexa Fluor 488 donkey anti-mouse IgG and/or Alexa Fluor 555 goat anti rabbit IgG, (Life Technologies- Invitrogen, Carlsbad, CA, USA) for 2 hours at RT. Finally, cells were stained with Hoechst 33258 (Life Technologies- Invitrogen, Carlsbad, CA, USA) (1∶5000 in DPBS) for 5 minutes, and maintained in PBS for epifluorescence microscopy.

### STED microscopy

For STED microscopy, H4 cells were plated in 15 mm coverslips. Forty-eight hours after transfection, the cells were washed with PBS and fixed with 4% PFA for 30 minutes at RT. The cells were rinse with quenching buffer (PBS +100 mM of Ammonium Chloride (Sigma-Aldrich, St. Louis, MO, USA)) for 15 minutes. After washing, the cells were permeabilized and blocked (2% BSA and 0.1% Triton X-100, in PBS) for 20 minutes. Cells were then incubated with primary antibodies against ASYN (1∶2000, BD Transduction Laboratory, New Jersey, USA) in the permeabilization-blocking solution diluted 1∶2 with PBS for 1 hour at RT. Finally, we incubated the cells with a secondary goat anti-mouse antibody labeled with Atto 647N (5 µg/mL; Sigma-Aldrich, St. Louis, MO, USA). STED images were taken with a Leica TCS STED system (Leica Microsystems) with a 100X oil objective (1.4 numerical aperture, NA, 1003 HCX PL APO CS oil; Leica Microsystems). For excitation, we used a 635 nm diode laser, and for the depletion donut (130 mW with the 1003 objective) we used a Spectra-Physics MaiTai multiphoton laser at 750 nm (Newport Spec- tra-Physics). Scans were performed at 1,000 Hz and the final images represent an average of 96 scans (STED) (average performed line-by-line) with the pinhole set at 47 µm. STED images were obtained at 20.20×20.20 nm pixels.

### Thioflavin S staining

After staining with secondary antibody, cells were incubated with freshly prepared 0.05% Thioflavin S (Sigma-Aldrich, St. Louis, MO, USA) for 5 minutes. Cells were then washed with 80% EtOH for 5 minutes and, finally, stained with Hoechst 33258, washed and maintained in PBS for fluorescence microscopy.

### Quantification of nuclear and cytoplasmic fluorescence intensities

Nuclear and cytoplasmic fluorescence intensities were quantified using ImageJ software (http://rsbweb.nih.gov/ij/). Using the freehand tool the nucleus and cytosol were selected and the respective intensities were measured. The results reflect the counting of 25 cells per experiment.

### Quantification of ASYN inclusions

Transfected cells were detected and scored based on the ASYN inclusions pattern and classified into four groups: cells without inclusions, less than five inclusions (<5 inclusions), between five to nine inclusions (≥5–9 inclusions) and more than ten inclusions (≥10 inclusions). Results were expressed as the percentage of the total number of transfected cells obtained from three independent experiments for each mutation.

### Quantification of Golgi fragmentation

Transfected HEK and H4 cells were scored based on the morphology of the Golgi apparatus and classified into three groups: non-fragmented, diffused and fragmented. Results were expressed as the percentage of the total number of transfected cells. Three independent experiments were performed.

### Western blot analyses

HEK and H4 cells were lysed with Radio-Immunoprecipitation Assay (RIPA) lysis buffer (50 mM Tris pH 8.0, 0.15 M NaCl, 0.1% SDS, 1% NP40, 0.5% Na-Deoxycholate), 2 mM EDTA and a Protease Inhibitor Cocktail (1 tablet/10 mL) (Roche Diagnostics, Mannheim, Germany). To detect phosphorylated-ASYN was added Phosphatase Inhibitor Cocktail (1 tablets/10 mL) (Roche Diagnostics, Mannheim, Germany). Protein concentration was determined using the Bradford assay (BioRad Laboratories, Hercules, CA, USA) and the gels were loaded with 40 µg protein after denaturation for 10 minutes at 100°C in protein sample buffer (125 mM of 1 M Tris HCl pH 6.8, 4% SDS 0,5% Bromphenol blue, 4 mM EDTA 20% Glycerol 10% β-Mercapto ethanol).

The samples were separated on 12% SDS-polyacrylamide gels (SDS-PAGE) with a constant voltage of 110 V using Tris-Glycine SDS 0.5% running buffer (250 mM Tris, 200 mM Glycin, 1% SDS, pH 8.3) for 60 minutes.

The transfer was carried out to nitrocellulose membrane (Protran, Schleicher and Schuell, Whatman GmbH, Dassel, Germany) for 90 minutes with constant current at 0.3 A using Tris-Glycine transfer buffer.

Membranes were blocked with 5% (w/v) skim milk (Fluka, Sigma-Aldrich, St. Louis, MO, USA) in 1xTBS-Tween (50 mM Tris, 150 mM NaCl, 0.05% Tween, pH 7.5) for 60 minutes at RT.

Membranes were further incubated with the primary antibody, either mouse anti-ASYN (1∶1000, BD Biosciences, San Jose, CA, USA) or rabbit anti-ASYN (1∶1000, Santa Cruz Biotechnologies, Santa Cruz, CA, US), anti-BiP (BD Biosciences, San Jose, CA, USA) and 1∶1000 mouse anti-β-actin (Sigma-Aldrich, St. Louis, MO, USA) in 3% Albumin Bovine Fraction V (BSA)/TBS-Tween (NZYTech, Lisbon, Portugal), at RT for 3 hours or overnight at 4°C.

After washing three times in TBS-Tween for five minutes, the membranes were incubated for 1 hour with secondary antibody, anti-mouse IgG, or anti-rabbit IgG, horseradish peroxidase labeled secondary antibody (GE Healthcare, Bucks, UK) at 1∶10000 in 3% milk/TBS-Tween.

Detection was done using Luminol Reagent and Peroxide Solution (Millipore, Billerica, MA, USA) and applied to the membrane 1 minute before scanning with in AlphaImager FluoroChem software (AlphaInnotech).

Protein levels were quantified using ImageJ and normalized to the β-actin levels.

H4 cells transfected with ASYN-hGLuc constructs were washed with cold PBS to remove excess cell culture media. Cell lysis buffer (20 mM NaCl, 0,6% Deoxycholate, 0,6% Igepal, 25 mM Tris pH 8.0, Protease Inhibitor Cocktail tablet, 1 tablet/10 mL) was added on human H4 cells in 60 mm dishes and incubated on ice for 10 minutes. After cell scraping samples were centrifuged for 10 minutes at 13,000 g. Lysates were resolved by electrophoresis on a 4–12% Bis-Tris gradient gel (NuPAGE Novex Bis-Tris Gel, Life Technologies, Darmstadt, Germany) according to manufacturer's instructions using NuPAGE MOPS buffer. After transfer to nitrocellulose membrane (Protran, Schleicher and Schuell, Whatman GmbH, Dassel, Germany) membranes were blocked in 1x Roti Block (Roth, Carlsbad, Germany) for 1 hour at RT. The forward steps were similar as describe above.

### Assessment of ASYN phosphorylation

The blots were directly removed from the blotting chamber and fixed in 0.4% PFA in PBS for 30 minutes. The membranes were briefly boiled in PBS, washed very short in TBS-Tween with phosphatase inhibitor (25 mM B-Glycerolphosphat, 5 mM NaF, 1 mM Na_3_VO_4_), and then were blocked in 5% BSA/TBS-Tween with phosphatase inhibitor for at least 1 hour (in the cold room). Anti-S129 phosphorylation ASYN 1∶1000, WAKO, Richmond, Virginia, USA) was prepared in 5% BSA/TBS-Tween with phosphatase inhibitor and incubated overnight in the cold room. After washing tree times with TBS-Tween with phosphatase inhibitor for 10 minutes, the membranes were incubated with secondary antibody (anti-mouse IgG) for 1,5 hours at RT. Finally they were washed and revealed as previously described.

### Native PAGE

For native PAGE, samples were lysed with detergent-free lysis buffer (50 mM Tris HCl pH 7.4, 175 mM NaCl, 5 mM EDTA pH 8.0 and Protease Inhibitor Cocktail tablet, 1 tablet/10 mL). Native-PAGE was run with detergent-free Tris-Glycine running buffer (250 mM Tris and 200 mM Glycin) and in protein sample buffer (1 M Tris HCl pH 6.8, Glycerol 100%, 0,4% Bromophenol blue).

### Detergent solubility experiments

The H4 cells were plated and transfected as previously describe. Using 80 µL lysing buffer 1 (25 mM Tris pH 7.5, 150 mM NaCl, 1 mM EDTA, 1% Triton X-100, ½ tablet of EDTA Protease Inhibitor) the cells were harvested and centrifuged at 100.000 g for 30 minutes at 4°C. Supernatants were collected (soluble fraction) and the pellets (insoluble fraction) were washed with cold PBS and transferred to new tubes.

Once again, the samples were centrifuged at 14.000 rpm for 10 minutes at 4°C and the pellets were resuspended in 50 µL lysing buffer 2 (75 mM Tris pH 6.8, 3% SDS, 15% Glycerin, 3.75 mM EDTA pH 7.4). Finally, the samples were sonicated (10 pulse/second) and a western blot was run as previously described.

The insoluble fraction was calculated as: 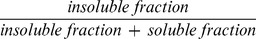 and then normalized for ASYN WT.

### Flow cytometry

HEK cells were plated and imaged after twenty-four hours, as described above. Then, cells were trypsinized, neutralized with growth medium, centrifuged (1500 g at 5°C) and the pellet was reconstituted in 7-aminoactinomycin D (7-AAD, Life Technologies- Invitrogen, Carlsbad, CA, USA) prepared 1∶1000 in PBS. The fluorescence was measured using a microcapillary system (GuavaeasyCyte HT system, Millipore). 25000 events were counted per sample.

### ASYN levels in the culture medium

A detailed description of the assay procedure has been published before [50]. [50]96-well Multi-Array standard plates (Meso Scale Discovery, Gaithersburg, MD, USA) were coated with 30 µl of MJF1 clone 12.1 (kindly provided by Liyu Wu, Epitomics, Burlingame, CA, USA) as capture antibodies at 3 µg/ml dissolved in PBS buffer and incubated overnight at 4°C without shaking. All washing steps were done three times with 150 µl PBS-T (PBS supplemented with 0.05% Tween-20). After washing away the capture antibodies plates were blocked with 1% BSA/PBS-T for 1 h at RT with shaking at 300 rpm. Standards and biosamples collected from H4 cells were diluted in 1% BSA/PBS-T and applied in 25 µl volumes. Incubation was done for 1 h (RT, 700 rpm). Plates were washed again and 25 µl Sulfo-TAG labelled ASYN (BD Biosciences, Heidelberg, Germany) were applied at 1 µg/ml. After a final washing 150 µl 2x Read Buffer T (MSD) were applied to the wells and plates were read in a Sector Imager 6000 (MSD).

### Toxicity assays – LDH release

For lactate dehydrogenase (LDH) cytotoxicity assay (Roche Diagnostics, Mannheim, Germany) the reaction mixture were prepared according to the manufacturer. The growth media from H4 cells were plated in triplicates in a 96 well plate, in a ratio 1∶1 with the reaction mixture. The absorbance measurements were performed in a TECAN Infinite 200 Pro plate reader at 490 nm. To determine the percentage cytotoxicity, the average absorbance values were subtracted with the average absorbance value obtained in the background control. The percentage of toxicity was calculated as indicated by the manufacturer.

### Statistical analyses

Data were analysed using GraphPad Prism 5 (San Diego California, USA) software and were expressed as the mean ± SEM of at least three independent experiments. The values of ASYN mutations from flow cytometry were normalized to WT ASYN and mean values for each experiment were determined. Statistical differences from WT ASYN were calculated using unpaired Student *t*-test. Significance was assessed for p≤0.05, where * corresponds to p≤0.05, ** corresponds to p≤0.01 and *** corresponds to p≤0.001.

## Supporting Information

Table S1 Primers used in the site directed mutagenesis. Primer used to performed site-directed mutagenesis and generated all the mutants versions of ASYN used in this study.
(PDF)

Table S2 Summary of the effects of the mutations on ASYN oligomerization and aggregation. The table resume the effects that each mutation had in the two systems analyze regarding the WT. In grey it is highlight the mutations that we used for further analysis.
(PDF)

Figure S1 Statistical analysis of ASYN inclusion formation. A. Cells without inclusions. B. Cells with less than five inclusions. C. Cells with more than 5 and less than 10 inclusions. D. Cells with more than ten inclusions. Student's *t* test (*p<0.05, **p<0.01, ***p<0.001). n = 3.
(PDF)

Figure S2 Morphological analysis of Golgi apparatus. A. Oligomerization paradigm B. Aggregation model paradigm. Student's *t* test (*p<0.05, **p<0.01, ***p<0.001). n = 3.
(PDF)

Figure S3 Phosphorylation state of ASYN on S129. A-B Phosphorylation of ASYN in the BiFC assay. E35K and E57K showed an increase of S129 ASYN phosphorylation. Student's *t* test (*p<0.05, **p<0.01, ***p<0.001). n = 3. B. Levels of ASYN S129 phosphorylation in the aggregation model.
(PDF)
