# Supplementary material for: Systematic Comparison of the Effects of Alpha-synuclein Mutations on Its Oligomerization and Aggregation
Source: PLoS Genet. 2014 Nov 13;10(11):e1004741. doi: 10.1371/journal.pgen.1004741 (PMC4230739; doi:10.1371/journal.pgen.1004741)
Supplement: Table S1 — Primers used in the site directed mutagenesis. Primer used to performed site-directed mutagenesis and generated all the mutants versions of ASYN used in this study. (PDF) [file pgen.1004741.s001.pdf]

Mutation	Primer
**A30P**	Forward: 5'-GGGTGTGGCAGAAGCACCAGGAAAGACAAAAGA-3'<br>Reverse: 5'-TCTTTTGTCTTTCCTGGTGCTTCTGCCACACCC-3'
**E46K**	Forward: 5'-TAGGCTCCAAAACCAAGAAGGGAGTGGTGCATGG-3’<br>Reverse: 5'-CCATGCACCACTCCCTTCTTGGTTTTGGAGCCTA-3’
**H50Q**	Forward: 5'-GGAGGGAGTGGTGCAGGGTGTGGCAACAG-3’<br>Reverse: 5'- CTGTTGCCACACCCTGCACCACTCCCTCC-3’
**G51D**	Forward: 5'-GGGAGTGGTGCATGATGTGGCAACAGTGG-3’<br>Reverse: 5'-CCACTGTTGCCACATCATGCACCACTCCC-3’
**A53T**	Forward: 5'-GAGTGGTGCATGGTGTGACGACAGTGGCTGAGAAGAC-3'<br>Reverse: 5'-GTCTTCTCAGCCACTGTCGTCACACCATGCACCACTC-3'
**E35K**	Forward: 5'-CAGAAGCAGCAGGAAAGACAAAAAAGGGTGTTCTCT-3'<br>Reverse: 5'-AGAGAACACCCTTTTTTGTCTTTCCTGCTGCTTCTG-3'
**E57K**	Forward: 5' GTGGCAACAGTGGCTAAGAAGACCAAAGAGC 3'<br>Reverse: 5' GCTCTTTGGTCTTCTTAGCCACTGTTGCCAC 3'
**A56P**	Forward: 5'-GGTGTGGCAACAGTGCCTGAGAAGACCAAAG-3'<br>Reverse: 5'-CTTTGGTCTTCTCAGGCACTGTTGCCACACC-3'
**A76P**	Forward: 5'-TGACGGGTGTGACACCAGTAGCCCAGAAG-3'<br>Reverse: 5'-CTTCTGGGCTACTGGTGTCACACCCGTCA-3'
**S129A**	Forward: 5'CTTATGAAATGCCTGCTGAGGAAGGGTATC-3’<br>Reverse: 5'GATACCCTTCCTCAGCAGGCATTTCATAAG-3’
**S129D**	Forward: 5'-GGCTTATGAAATGCCTGATGAGGAAGGGTATCAAG-3’<br>Reverse: 5'-CTTGATACCCTTCCTCATCAGGCATTTCATAAG CC-3’
**S129G**	Forward: 5'-GACAATGAGGCTTATGAAATGCCTGGTGAGGAAGGGTATC-3'<br>Reverse: 5'-GATACCCTTCCTCACCAGGCATTTCATAAGCCTCATTGTC-3'
**S87A**	Forward: 5'-AAGACAGTGGAGGGAGCAGGGGCCATTGCAGCAG-3'<br>Reverse: 5'-CTGCTGCAATGGCCCCTGCTCCCTCCACTGTCTT-3'
**S87E**	Forward: 5'-ACAGTGGAGGGAGCAGGGGAAATTGCAGCAGC-3'<br>Reverse: 5'-GCTGCTGCAATTTCCCCTGCTCCCTCCACTGT-3'
**Y125F**	Forward: 5'-GGATCCTGACAATGAGGCTTTTGAAATGCCTTCTGA-3'<br>Reverse: 5'-TCAGAAGGCATTTCAAAAGCCTCATTGTCAGGATCC-3'
**Y125D**	Forward: 5'-GATCCTGACAATGAGGCTGATGAAATGCCTTCTGAGG-3'<br>Reverse: 5'-CCTCAGAAGGCATTTCATCAGCCTCATTGTCAGGATC-3'
**K96R**	Forward: 5'-GCCACTGGCTTTGTCAGAAAGGACCAGTTGGGC-3'<br>Reverse: 5'-GCCCAACTGGTCCTTTCTGACAAAGCCAGTGGC-3'
**K102R**	Forward: 5'-AAGGACCAGTTGGGCAGGAATGAAGAAGGAGCC-3'<br>Reverse: 5'-GGCTCCTTCTTCATTCCTGCCCAACTGGTCCTT-3'
