# Supplementary material for: Systematic Comparison of the Effects of Alpha-synuclein Mutations on Its Oligomerization and Aggregation
Source: PLoS Genet. 2014 Nov 13;10(11):e1004741. doi: 10.1371/journal.pgen.1004741 (PMC4230739; doi:10.1371/journal.pgen.1004741)
Supplement: Table S2 — Summary of the effects of the mutations on ASYN oligomerization and aggregation. The table resume the effects that each mutation had in the two systems analyze regarding the WT. In grey it is highlight the mutations that we used for further analysis. (PDF) [file pgen.1004741.s002.pdf]

Mutation	Effects on oligomerization			Effects on aggregation	
	Flow Cytometry	Subcellular distribution		Number	Morphology
		Nucleus	Cytosol		
**A30P**	↔	↑	↓	↓	↔
**E46K**	↔	↔	↔	↑	Mix
**A53T**	↔	↑	↓	↔	↔
**H50Q**	↔	↑	↓	↑	↔
**G51D**	↔	↑	↓	↑	↔
**A56P**	↑	↔	↔	↓	↔
**A76P**	↑	↔	↔	↓	↔
**A30P/A76P**	↑	↑	↓	↓	↔
**TP**	↑	↔	↔	↓	↔
**E35K**	↑	↔	↔	↑	Mix
**E57K**	↑	↔	↔	↑	Small
**S87A**	↔	↔	↔	↔	↔
**S87E**	↓	↔	↔	↓	↔
**K96R/K102R**	↑	↔	↔	↔	Mix
**Y125D**	↔	↔	↔	↔	↔
**Y125F**	↔	↔	↔	↔	Mix
**S129A**	↔	↑	↓	↔	↔
**S129G**	↔	↔	↔	↔	↔
**S129D**	↔	↔	↔	↔	↔
