# Supplementary figures and images for: Systematic Comparison of the Effects of Alpha-synuclein Mutations on Its Oligomerization and Aggregation
Source: PLoS Genet. 2014 Nov 13;10(11):e1004741. doi: 10.1371/journal.pgen.1004741 (PMC4230739; doi:10.1371/journal.pgen.1004741)

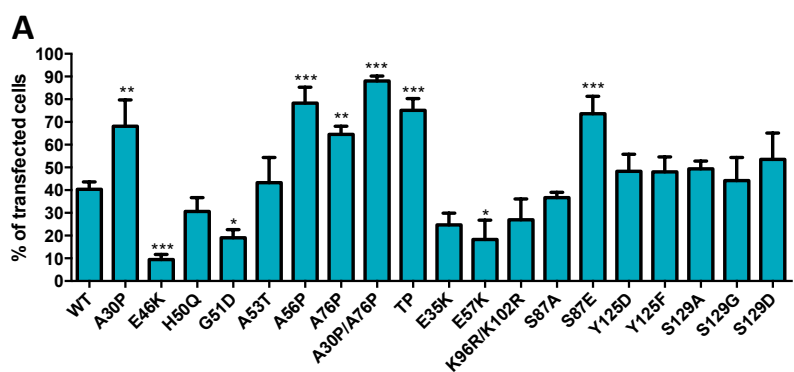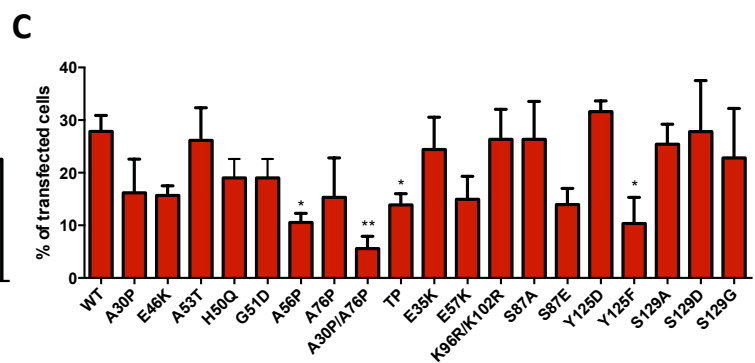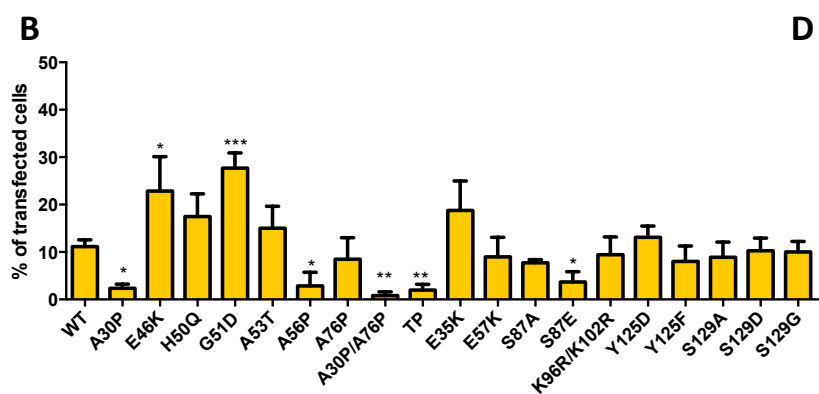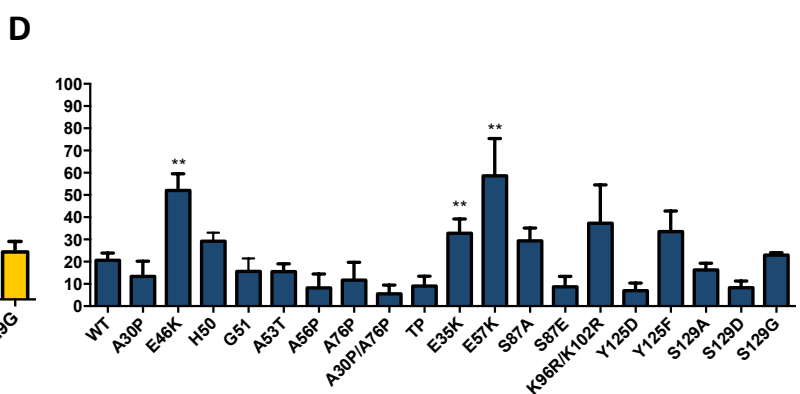

Supplement: Figure S1 — Statistical analysis of ASYN inclusion formation. A. Cells without inclusions. B. Cells with less than five inclusions. C. Cells with more than 5 and less than 10 inclusions. D. Cells with more than ten inclusions. Student's t test (*p<0.05, **p<0.01, ***p<0.001). n = 3. (PDF) [file pgen.1004741.s003.pdf]

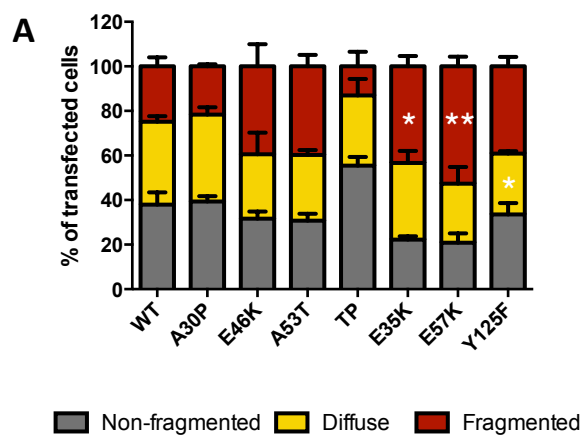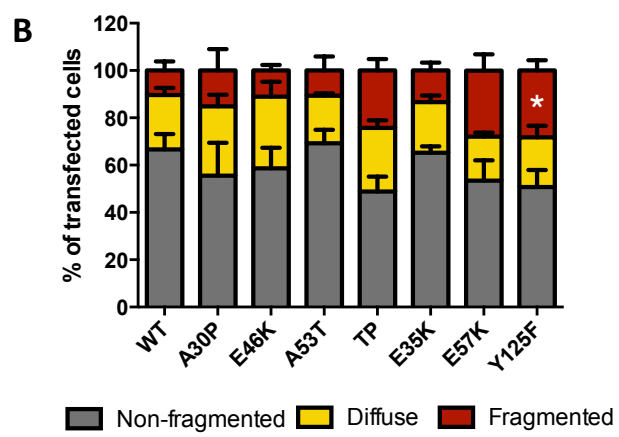

Supplement: Figure S2 — Morphological analysis of Golgi apparatus. A. Oligomerization paradigm B. Aggregation model paradigm. Student's t test (*p<0.05, **p<0.01, ***p<0.001). n = 3. (PDF) [file pgen.1004741.s004.pdf]

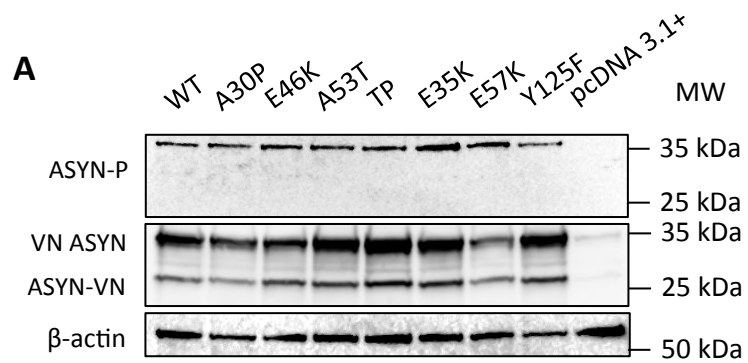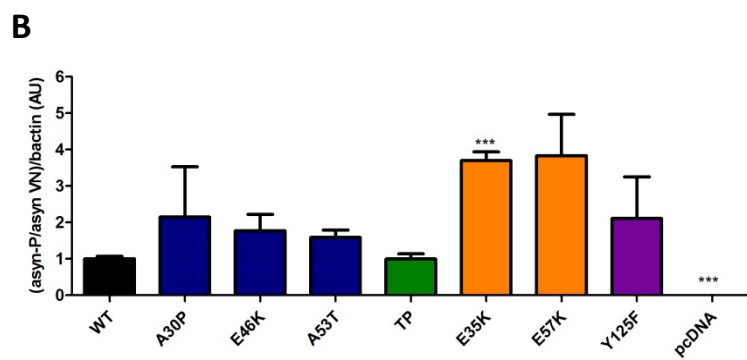

Supplement: Figure S3 — Phosphorylation state of ASYN on S129. A-B Phosphorylation of ASYN in the BiFC assay. E35K and E57K showed an increase of S129 ASYN phosphorylation. Student's t test (*p<0.05, **p<0.01, ***p<0.001). n = 3. B. Levels of ASYN S129 phosphorylation in the aggregation model. (PDF) [file pgen.1004741.s005.pdf]
